# Pulmonary fibrosis on the lateral chest radiograph: Kerley D lines revisited

**DOI:** 10.1007/s13244-017-0565-2

**Published:** 2017-08-07

**Authors:** Daniel B. Green, Alan C. Legasto, Ian R. Drexler, James F. Gruden

**Affiliations:** Department of Radiology, New York-Presbyterian Hospital/Weill Cornell Medical College, 525 E. 68th St, Box 141, New York, NY 10065 USA

**Keywords:** Pulmonary fibrosis, Lung diseases, interstitial, Thoracic radiography, Multidetector computed tomography, Pulmonary emphysema

## Abstract

**Abstract:**

The retrosternal clear space (RCS) is a lucent area on the lateral chest radiograph located directly behind the sternum. The two types of pathology classically addressed in the RCS are anterior mediastinal masses and emphysema. Diseases of the pulmonary interstitium are a third type of pathology that can be seen in the RCS. Retrosternal reticular opacities, known as Kerley D lines, were initially described in the setting of interstitial oedema. Pulmonary fibrosis is another aetiology of Kerley D lines, which may be more easily identified in the RCS than elsewhere on the chest radiograph.

***Teaching points*:**

*• The RCS is one of three lucent spaces on the lateral chest radiograph.*

*• Reticular opacities in the RCS are known as Kerley D lines.*

*• Pulmonary fibrosis can be seen in the RCS as Kerley D lines.*

*• Kerley D lines should be further evaluated with chest CT.*

## Introduction

**Fig. 1:**
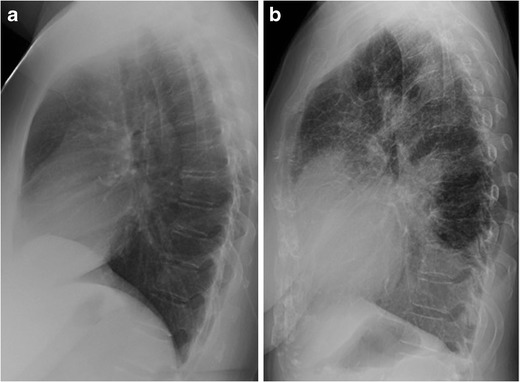
Lateral chest radiographs demonstrating a normal retrosternal clear space (a), the lucent area between the sternum and ascending aorta, and retrosternal reticular opacities due to interstitial pulmonary oedema, known as “Kerley D lines” (b). Pleural effusions accompany the oedema

The retrosternal clear space (RCS), or retrosternal air gap, is one of three “clear spaces” on the lateral chest radiograph, along with the retrocardiac clear space and the retrotracheal clear space. It is a lucent area located between the sternum and ascending aorta, above the heart border formed by the right ventricular outflow tract [1] (Fig. 1).

**Fig. 2:**
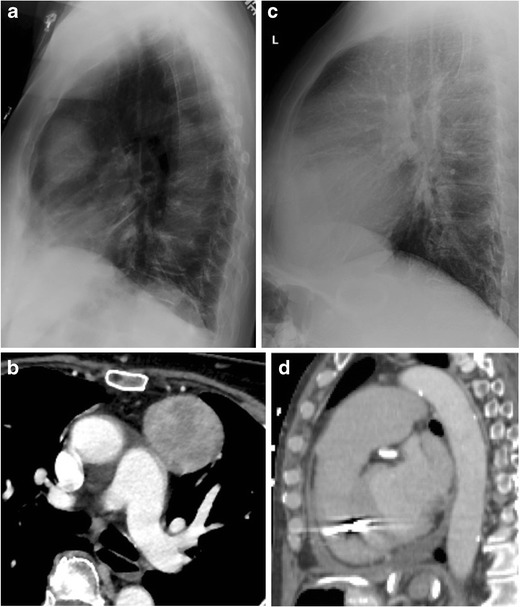
Two examples of classic retrosternal clear space pathology: a lateral chest radiograph (a) and axial CT (b) showing an anterior mediastinal mass, and a lateral chest radiograph (c) and sagittal CT (d) showing an enlarged right ventricle and main pulmonary artery in a patient with pulmonary hypertension

The classic teaching is that the RCS depicts two types of pathology. Increased opacity within the RCS signifies the presence of either an anterior mediastinal mass or an enlarged right ventricle (Fig. 2), and an expanded RCS can be seen in obstructive lung diseases such as emphysema [2–4].

Diseases of the pulmonary interstitium are another type of pathology that can be identified in the RCS. Owing to its lucency, the RCS clearly illustrates reticular opacities that may be obscured in other regions of the chest radiograph. Kreel et al. (1975) first described reticular opacities in the RCS in the setting of interstitial pulmonary oedema before CT was readily available [5]. They named these septal lines “Kerley D lines,” expanding upon Kerley’s initial characterization of A, B, and C lines on the frontal radiograph. Linear opacities in the periphery of the lung that extend to the pleural surface, Kerley D lines have the same appearance as the more familiar Kerley B lines, but in a different location (Fig. 1b).

We present a case-based review of pulmonary fibrosis detected in the RCS—analogous to Kerley D lines caused by interstitial oedema—with CT correlation.

Although high-resolution chest CT is the gold standard for evaluating interstitial lung disease, chest radiography is frequently the initial investigation, despite its limitations. A confident diagnosis of the aetiology of pulmonary fibrosis can be made in only 23% of cases on radiography [6], and up to 10% of patients with pulmonary fibrosis have a normal chest radiograph with normal lung volumes [7]. Early interstitial lung disease may also be an incidental finding in patients with subclinical or undiagnosed fibrosis. This highlights both the difficulty and importance of detecting interstitial lung disease on chest radiography.

## Discussion

**Fig. 3:**
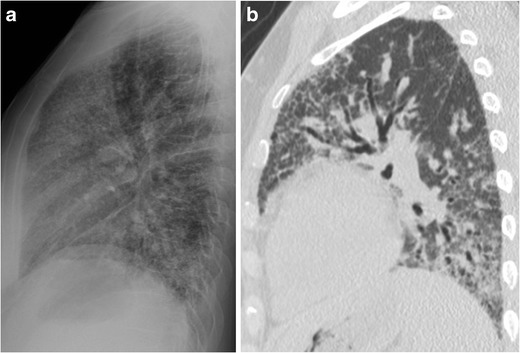
53-year-old woman with chronic hypersensitivity pneumonitis. The lateral chest radiograph (a) projects fibrosis as well through the retrosternal clear space as at the lung bases. A corresponding sagittal CT slice (b) is also shown

**Fig. 4:**
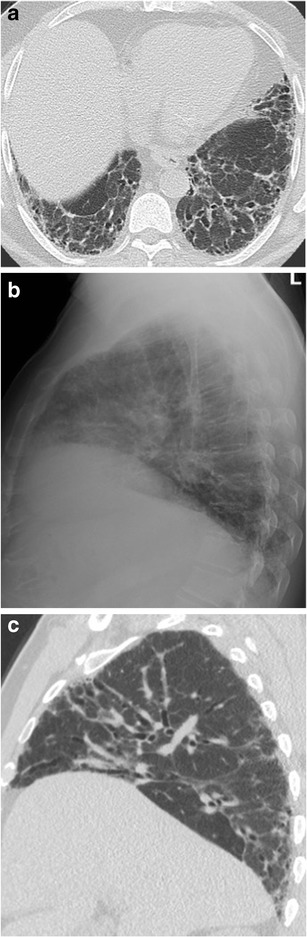
68-year-old man with idiopathic pulmonary fibrosis. An axial CT slice (a) shows fibrosis at the lung bases. On the lateral radiograph (b), reticular opacities are seen in the retrosternal clear space but not at the lung bases, where they are obscured by pulmonary vasculature and the spine. A corresponding sagittal CT slice (c) is also shown

Reticular opacities due to interstitial lung disease may be most easily identified on the lateral chest radiograph in the RCS, and in some instances may only be identifiable in the RCS. Fibrosis patterns can be upper or lower lung-predominant, as well as either early- or late-stage disease. Since the RCS corresponds to the anterior upper lobes, it is not surprising that an upper lung-predominant fibrosis such as chronic hypersensitivity pneumonitis would be detectable (Fig. 3). It is less intuitive, however, that a basilar-predominant fibrosis could be most conspicuous in the RCS, until considering that some anterior upper lobe involvement is a well-documented, typical feature of usual interstitial pneumonia (UIP; Fig. 4) [8]. We have not encountered other causes of upper lung fibrosis, such as sarcoidosis or pleuroparenchymal fibroelastosis, in the RCS.

**Fig. 5:**
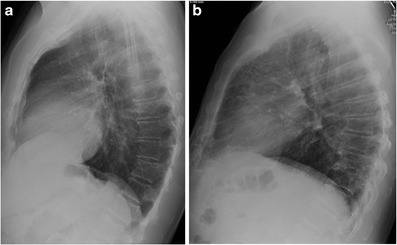
63-year-old asymptomatic man with a normal radiograph (a) showing fine linear opacities in the retrosternal clear space that taper before reaching the sternum. In contrast, coarse reticular opacities form a mesh and extend to the sternum (b) in a 67-year-old man with an unclassified pulmonary fibrosis (CT not shown)

Several factors limit the detection of reticular opacities in other regions of the chest radiograph. On the frontal view, atelectasis and normal pulmonary vasculature are also linear and can obscure septal lines. On the lateral view, normal pulmonary vasculature and the spine are superimposed on the lower lobes (Fig. 4). Conversely, the RCS is devoid of overlapping structures. Normal pulmonary vessels in the retrosternal region are peripheral and often too fine to be seen. As a result, reticular opacities in the RCS may be the only discernible evidence of interstitial lung disease on chest radiography. Fine linear opacities due to normal vasculature may be seen in the RCS, but these lines eventually taper off, whereas abnormal reticular opacities are coarser, create a mesh-like appearance, and extend to the sternum (Fig. 5).

**Fig. 6:**
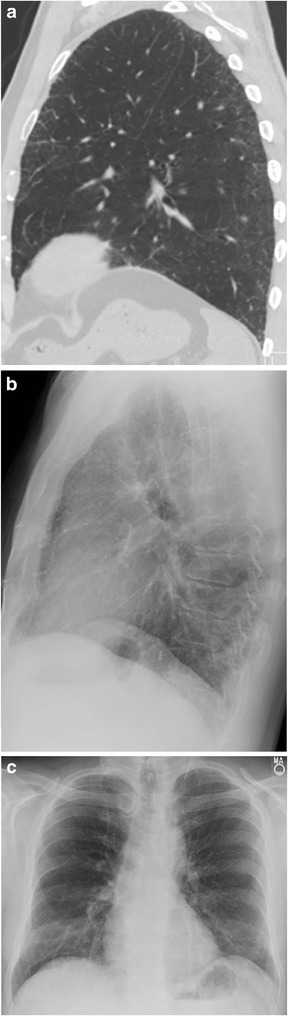
67-year-old man with mild pulmonary fibrosis anteriorly and posteriorly on a sagittal CT slice (a). On the lateral radiograph (b), fibrosis is only evident anteriorly, despite the small size of the retrosternal clear space. The frontal radiograph (c) is normal

**Fig. 7:**
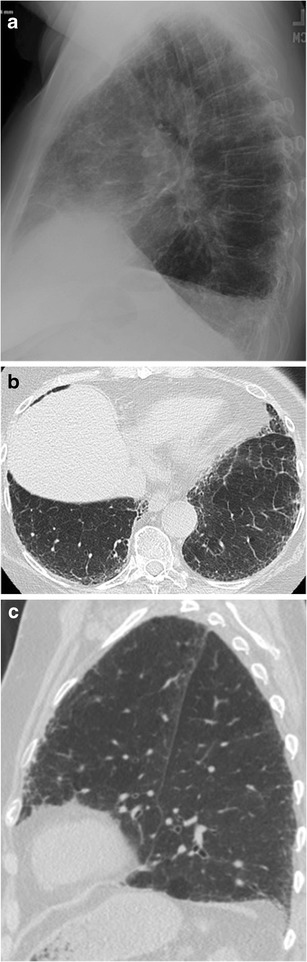
84-year-old woman with idiopathic pulmonary fibrosis. On the lateral radiograph (a), reticular opacities are easily seen through an opaque retrosternal space. Although the retrocardiac space is more lucent, reticular opacities are not seen at the lung bases, despite the basilar predominance typical of UIP demonstrated on axial (b) and sagittal (c) CT slices

Some individuals with an abundance of soft tissue in the chest wall, such as obese people or women with large breasts, have an opaque retrosternal space. In these instances, the opacity of the retrosternal region is similar to that of the heart and not as lucent as the retrocardiac clear space [9]. Nevertheless, basilar-predominant pulmonary fibrosis may still be better visualized in an opaque RCS than in the retrocardiac clear space (Figs. 6 and 7).

Although a lateral radiograph is not routinely performed at some institutions, nearly all of the outpatient chest radiographs at our institution include both frontal and lateral views. Many of our referrers request frontal and lateral radiographs as the initial work-up for interstitial lung disease prior to chest CT. We have found the lateral view to be a useful supplement to the frontal radiograph, particularly for evaluation of the RCS. In addition to occasionally being the only sign of interstitial lung disease on otherwise normal radiographs, the recognition of Kerley D lines can also boost the confidence of the radiologist who suspects but is uncertain about reticular opacities on the frontal view.

**Fig. 8:**
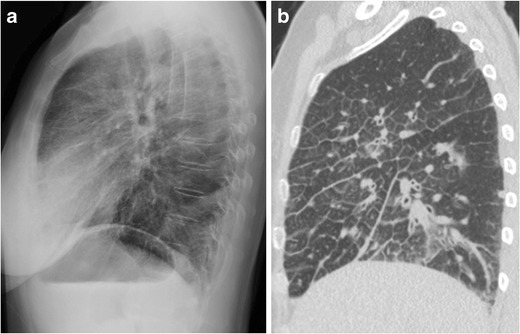
62-year-old woman with metastatic lung adenocarcinoma and lymphangitic carcinomatosis. Interlobular septal thickening is clearly seen in the retrosternal clear space on the lateral radiograph (a). A corresponding sagittal CT slice (b) is also shown

**Fig. 9:**
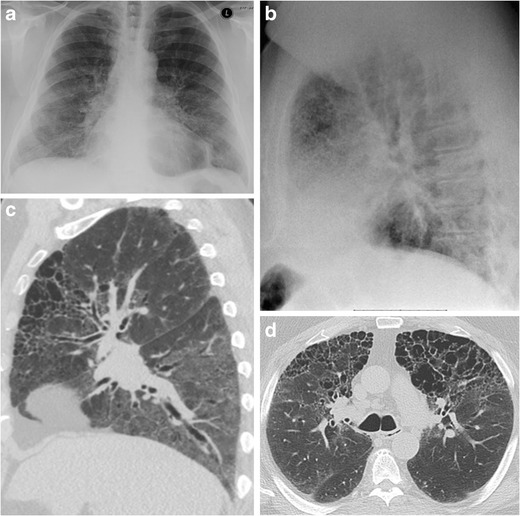
55-year-old man with smoking history. The frontal radiograph (a) is normal, and the lateral radiograph (b) shows reticular opacities in the retrosternal clear space only. Axial (c) and sagittal (d) CT slices show paraseptal and centrilobular emphysema corresponding to the radiographic abnormality. Ground-glass opacities in the lower lobes are suspected to be due to desquamative interstitial pneumonia

Other causes of reticular opacities in the RCS besides pulmonary fibrosis and interstitial pulmonary oedema include lymphangitic carcinomatosis (Fig. 8), viral pneumonia, and emphysema. Though emphysema is not a disease of the interstitium, reticular opacities corresponding to thickened walls of bullae can mimic interstitial lung disease (Fig. 9). We recommend that reticular opacities of any aetiology identified in the RCS be considered abnormal, and depending on the clinical scenario, should prompt further evaluation with chest CT. Attempting to characterise presumed fibrosis with radiography alone is not necessary and is best left for CT. On the other hand, suspected viral pneumonia or interstitial pulmonary oedema do not necessarily warrant further evaluation.

## Conclusion

Kerley D lines were initially described in regard to interstitial pulmonary oedema in the RCS on the lateral chest radiograph. Pulmonary fibrosis is another potential source of Kerley D lines, which may serve as an early indicator of interstitial lung disease on an otherwise normal exam. In institutions where frontal and lateral radiographs are routinely performed in the initial work-up for pulmonary fibrosis, we suggest close inspection of the RCS. When Kerley D lines are identified and pulmonary fibrosis is suspected, a chest CT should be obtained for further evaluation.
